# Technology supports me: Perceptions of the benefits of digital technology in adolescents

**DOI:** 10.3389/fpsyg.2022.970395

**Published:** 2023-01-30

**Authors:** Laura Bitto Urbanova, Andrea Madarasova Geckova, Zuzana Dankulincova Veselska, Silvia Capikova, Jana Holubcikova, Jitse P. van Dijk, Sijmen A. Reijneveld

**Affiliations:** ^1^Department of Health Psychology and Research Methodology, Faculty of Medicine, P. J. Safarik University, Kosice, Slovakia; ^2^Graduate School Kosice Institute for Society and Health, Faculty of Medicine, P. J. Safarik University, Kosice, Slovakia; ^3^Department of Community and Occupational Medicine, University Medical Center Groningen, University of Groningen, Groningen, Netherlands; ^4^Institute of Applied Psychology, Faculty of Social and Economic Sciences, Comenius University Bratislava, Bratislava, Slovakia; ^5^Institute of Social Medicine and Medical Ethics, Faculty of Medicine, Comenius University Bratislava, Bratislava, Slovakia

**Keywords:** qualitative study, digital technology, subjective perceptions, benefits, adolescents

## Abstract

**Background:**

Technology plays a significant role in the lives of adolescents. Our knowledge is predominantly based on research exploring the risks associated with it, but adolescents also feel that technology supports their lives. This has received less consideration. Therefore, we aim to examine how adolescents perceive the benefits of digital technology.

**Methods:**

We used qualitative data collected as part of the international Health Behaviour in School-Aged Children study. We conducted online, semi-structured interviews with 15 Slovak adolescents who came from three different types of secondary schools based on their graduation systems (mean age: 15.33; 20% boys). The data were analyzed using consensual qualitative research and thematic analysis.

**Results:**

We identified five main themes based on the comments of adolescents: 1. *I know* (source of information, formal and non-formal education); 2. *I can* (smart device, helpful tool); 3. *I am connected/included* (social interactions); 4. *I have my comfortable place* (leisure time, creating my alternative world); and 5. *I work on my future* (self-development).

**Conclusion:**

Adolescents perceived digital technology as mostly supportive and a helpful tool in their lives. The potential benefits of digital technology should be better reflected in public perception and policy, as the societal debate is mostly dominated by perceived disadvantages and risks.

## 1. Introduction

Technology is a very important element for young people, as it provides them with an alternative space where they can develop themselves and deal with different life challenges. Nowadays, the digital world is often perceived as a natural environment for adolescents, as they are used to working with it from an early age (Mascheroni and Ólafsson, 2014). In that world, digital technology helps them apply ICT (information and communication technology) for various purposes (Digital Technologies Hub, 2014; Smahel et al., 2020). Moreover, they belong to the most connected age group in the world (UNICEF for Every Child, 2017). On the one hand, the digital world offers them many opportunities for their personal development, but on the other hand, it can also bring many negative experiences that they have to deal with (Subrahmanyam and Smahel, 2010; Kalmus et al., 2014; Smahel et al., 2020).

The attention and evidence on the risks of digital technology are quite extensive, with evidence showing that adolescents face different types of challenges while using it. Examples include exposure to inappropriate content (sexual or aggressive content) and inappropriate behavior (online harassment, online grooming, cyberbullying, hacking, or sharing personal information), and technical issues like poor Wi-Fi and other connections (Best et al., 2014; Machimbarrena et al., 2018; Smahel et al., 2020). Excessive Internet use is another problem linked to digital technology, which can also have a negative impact on adolescents’ mental (depression, anxiety, stress, social isolation) and physical (headaches, backaches, dry eyes) health (Morrison and Gore, 2010; Koc, 2011; Best et al., 2014; Zheng et al., 2016; Guzel et al., 2018). However, digital technology can also be a very useful tool in the lives of adolescents if it is used appropriately. Their digital literacy, or ability to use digital technology for competent, autonomous, and safe communication, plays a crucial role in this process (Mascheroni and Ólafsson, 2014).

Based on theory and past research, digital technology seems to have several advantages for adolescents; however, evidence of their perspectives on this issue is lacking. First, the Co-construction model, proposed by Greenfields (1984) and adapted by Subrahmanyam and Smahel (2010), states that adolescents are no longer passive users of digital devices and virtual reality. On the contrary, in collaboration with other users, they participate in its co-construction, e.g., by creating and sharing norms that are in accordance with the properties of virtual space and by deciding the functions of applications or platforms (Greenfield and Yan, 2006). Regarding this, the boundaries between the online and offline worlds are disappearing for them. Moreover, the digital world serves as an extension of the offline world for adolescents. To be more specific, it allows them to address some offline issues or key developmental aspects related to their sexuality, identity, intimacy, or interpersonal relationships, for example by providing them with a vast amount of information that aids in their understanding of such offline experiences (Subrahmanyam and Smahel, 2010). In connection with this, findings of the Health Behaviour in School-Aged Children (HBSC) study showed that almost 20% of Slovak adolescents found it easier to talk about their feelings or worries online than face-to-face (Madarasova Geckova and Dankulincova, 2019).

Secondly, digital technology also plays a crucial role in an adolescent’s process of self-construction or socialization (Schmitt et al., 2008; Guan and Subrahmanyam, 2009; Subrahmanyam and Smahel, 2010). Regarding social interactions, digital technology has been found to be a very beneficial tool for adolescents to strengthen their existing relationships (Subrahmanyam and Greenfield, 2008; Lee, 2009). Thanks to digital technology, adolescents can gain self-disclosure experience or positive feedback from other users, which can increase their self-esteem, self-acceptance, perceived social support, and social desirability (Best et al., 2014; Cipotella et al., 2020). In addition, research has shown that for specific groups of people, digital technology can be even more beneficial, e.g., it can empower youth in disadvantaged circumstances, such as hearing-impaired children (Barak and Sadovsky, 2008). However, further research is needed to identify other predisposing properties of adolescents who are more likely to benefit from digital technology.

Third, digital technology has a unique potential to help adolescents develop their knowledge, for example, by accessing huge amounts of information easily, sharing the information or allowing group work not only regarding education in school, but also in other life domains, e.g. health (health websites, Internet-based prevention programs) (Ybarara et al., 2008; Baheiraei et al., 2014; Costa et al., 2014). Moreover, technology allows adolescents to participate in virtual classrooms and online courses if they cannot attend classes in person for various reasons, such as distance, the Covid-19 pandemic, and so on (Rose et al., 2022). Using these functions of digital technology can improve adolescents’ technical skills, which can also boost their self-confidence. Regarding health, research has shown that the use of digital technology to obtain health information is associated with positive youth development (Gómez-Baya et al., 2022). A key role is played by the parents of these adolescents, who are significant role models in the use of digital technology (Gulec et al., 2022). Research has also shown that technology can be a very useful tool in sharing health information between adolescents, by inviting pediatric influencers who spread health messages to their followers (Bozzola et al., 2021). The digital world is not only a place where adolescents gain something; it also allows them to talk openly with a doctor about sensitive topics related to their health (Harvey et al., 2008). Moreover, many adolescents find it useful to use digital devices, such as smartphones, watches, and apps, for monitoring their health (physical activity, menstruation), and for supporting some of its aspects (mood-enhancement and skill building; Pretorius et al., 2019; Rich et al., 2020). However, there is a need for further research on the ways digital technology can be even more effective in promoting education and health.

However, most research on digital technology has focused on its risks. Evidence is mostly lacking on the benefits, and only a very few studies have addressed adolescents’ subjective experiences and perspectives regarding the benefits of digital technology in life domains other than interpersonal relationships. Therefore, our study aims to assess the perception of adolescents regarding the benefits of digital technology. Such evidence can be very useful in strengthening the confidence of people in the capacity of digital technology to develop or improve social connections, education, and even their health.

## 2. Methods

### 2.1. Design of the study

We conducted qualitative research embedded within the international HBSC study mapping health and health-related behavior with respect to the social context of adolescents. This qualitative study has been conducted using a combination of consensual qualitative research (CQR; Hill et al., 2005) and thematic analysis methodologies (Braun and Clarke, 2006).

The study protocol has been approved by the Ethics Committee of the Medical Faculty at Pavol Jozef Safarik University in Kosice (19 N/2020), and therefore the study has been conducted in accordance with the ethical standards outlined in the Declaration of General Assembly of the World Medical Association (2014) and the consolidated criteria for reporting qualitative research (COREQ; Tong et al., 2007).

### 2.2. Study setting, sampling, and participants

Our sample consisted of 15 students (mean age: 15.33 years; standard deviation: 0.62) who were attending the first year of secondary school. We performed the sampling for this qualitative study in multiple steps. Firstly, we contacted the school administrators to inform them about our study. After obtaining their consent for participation in the study, we contacted the parents of the potential participants and obtained their informed consent. If the parents agreed with their child’s involvement, we got in touch with the adolescents. Participation in the study was fully voluntary and confidential, and all respondents were allowed to withdraw from it at any time.

### 2.3. Procedures and measures

First, we asked adolescents to fill out a questionnaire focused on sociodemographic characteristics (age, gender, and size of the place of residence). We then conducted nine semi-structured individual or group interviews that were based on a topic guide consisting of the following set of basic digital technology-related questions:

1. *When and why do you start using your mobile phone or tablet, connect to the Internet, or get online?*
2. *How does the Internet make your life easier? How does it help you?*
3. *In what way can the Internet be dangerous for people?*
4. *How do you know when time spent on your mobile phone, tablet, or online has become excessive?*
5. *How should mobile phones, tablets, or the Internet improve to serve you in the best possible way, becoming something that helps you, thanks to which you feel better, or that helps you to get closer to your goals?*

Each interview lasted 45–60 min. The interviews were conducted in the Slovak language and done via the online platform Zoom, as they were conducted during the second wave of Covid-19 pandemic in Slovakia (winter 2020/2021) and due to government measures Slovaks did not have a chance to meet with participants face-to-face. Each interview was conducted by a trained professional in psychology who had previous experience working with adolescents on an online counseling platform; all the interviews were video recorded. The rest of the research team consisted of researchers with a background in psychology, and all of them participated in the interviews as silent observers.

### 2.4. Data handling and analysis

Regarding data handling, we processed the obtained data by transcribing the interviews verbatim into the Slovak language. The transcriptions were checked to ensure their accuracy and then uploaded to MAXQDA, the standard platform used for data analysis. Next, we coded the data using the CQR methodology and thematic analysis. The team of coders consisted of a lead investigator (AMG), a senior (ZD, SC), and junior researchers (LBU), all of whom were trained in the CQR methodology. Individually, each team member watched the video recordings, read the transcripts, and created codes for the transcript segments. After that, all members met and shared their codes and interpretations with the aim of achieving consensus. In cases where members’ opinions differed, the discussion continued until they reached a consensus regarding the codes.

In the analysis, we first described our study sample using the data from the questionnaires. We then went through the entire transcript using thematic analysis and identified the benefits of digital technology as perceived by adolescents. The codes produced during data handling were clustered into subthemes and themes. All team members first did this individually. They then met to share the created subthemes and themes and discussed these until they reached a consensus regarding the final thematic map.

## 3. Results

### 3.1. Description of the sample

Table 1 provides the background characteristics of the sample. We obtained responses from 15 adolescents, whose mean age was 15.3 years, and 20% of whom were boys. In terms of education, more than 70% of our respondents attended a secondary school with a General Certificate of Secondary Education (GCSE) graduation, and the rest of the sample attended a grammar school or secondary school without a GCSE.

**Table 1:** Descriptive characteristics of the sample.

**Gender**	
Boys	3
Girls	12
**Type of school**	
Grammar school	3
Secondary school (GCSE)	11
Secondary school (apprenticeship certificate)	1
**Age (mean, SD)**	15.33 (0.62)

GCSE, General Certificate of Secondary Education.

### 3.2. Main themes

**Figure 1:**
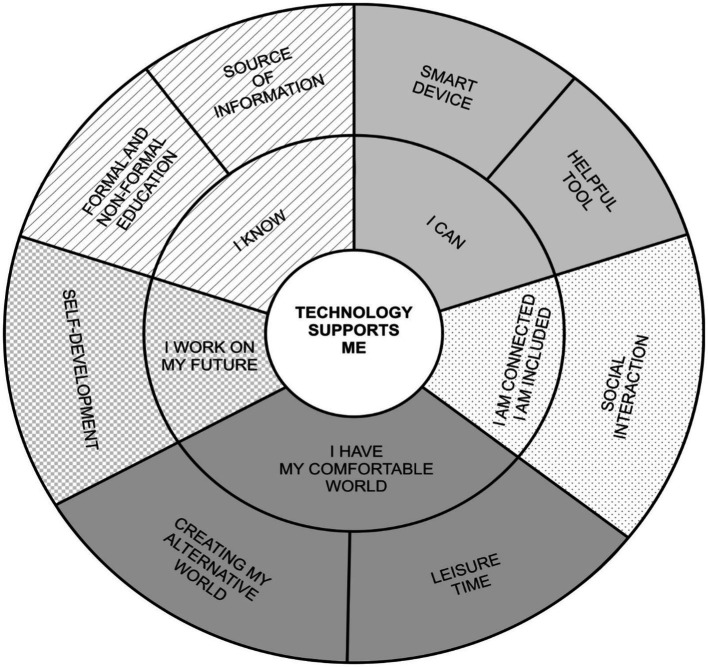
Model of themes and subthemes of perceived benefits of digital technology and Internet use by adolescents.

We identified five main themes regarding the different life domains in which digital technology can support and/or help adolescents, with each theme having several subthemes: 1. I know *(source of information, formal and non-formal education*); 2. I can (*smart device, helpful tool*); 3. I am connected/I am included (*social interactions*); 4. I have my comfortable world (*leisure time, creating my alternative world*); and 5. I work on my future (*self-development*), see Figure 1. Table 2 provides quotes regarding the mentioned themes.

**Table 2:** Selected participant quotations for identified themes and subthemes on the benefits of digital technology for adolescents.

Themes/ Subthemes	Quotations
I know	
*Source of information*	*“… or if you want to search for something, you have it right in your pocket, so you can find it.”*
	*“… in fact, it also helps at school, for example, we do not have to look for some information in different books, or something like that, we just write it on Google, and in a few seconds, we will actually find it. “*
*Formal & non-formal education*	*“For example, I play guitar, and I have watched a lot on the Internet about how I should learn to play some song or something, so it can help with many things…”*
	*“For example, there would be no courses, but some online classes outside of school about something that we are interested in or that we probably want to do in the future, or some course at school, but outside of the school schedule. Some course that we could take to learn something more than what we have in school.”*
I can	
*Smart device*	*“I would probably miss the phone the most because you can make calls and I think you can do the most things on the phone. It is the handiest. You can just take it everywhere.”*
	*“Seeing what we have at school, what I should do, what I should send. It is easier for me if I have all the information on my phone, or the Internet student book if I have everything written there. It’s easier than searching in a notebook, which we have to do for homework, which I should send to the teacher…”*
*Helpful tool*	*“Or for example, you can put some reminders on the phone, so we do not forget some important dates or meetings.”*
	*“… Also, in practical terms, when people have some problem, many tips or tricks can be found on the Internet, nearly for everything. When something at home needs to be repaired, or bought, or has a lot of recipes for cooking.*”
I am connected/I am included	
*Social interactions*	*“… that actually those important things for school, or as we mentioned last time, that in the evening when we want to chat with friends or call or spend some time with friends, or family, so I think that this is the reason why many people actually use the phone. Or when we want to go out, I think that the majority of people arrange meetings through social media.”*
	*“No, but I do not mean that I’m dependent on social media or something, but mainly for communication with other people. For example, if I do not understand something, for example, at school, I’ll write to someone to ask him/her if he/she can explain it to me. I know what is going on with that person; if I cannot be with him/her, the communication via phone tells me.”*
I have my comfortable world	
*Creating my alternative world*	*“For example, on Instagram or Facebook I have friends, or I follow people who motivate me in some way, or who are doing something similar that I would like to achieve, and I do not have people who demotivate me or who just talk about having a bad life and so on.”*
	*“It gives me a greater feeling of freedom, regarding information and communication, and I can see the benefits of it.”*
*Leisure time*	*“Usually now, when there is more work and there is nothing to do now, I always finish schoolwork in the evenings, and immediately I turn on something like ‘Discord’, so I can hear my friends and play something to switch off a little.”*
	*“Then, when I take some breaks during learning, I also use it as a form of relaxation, so I watch some videos or check new messages and respond to them or something like that. So, I use it as a form of entertainment as well.”*
I work on my future	
*Self-development*	*“So, if we see something on the Internet, for example, how other people work out, it can motivate us to do something, to do some exercise as well. So, I am not just watching other people do the activity, I am also doing it.”*
	*“Or you can create a lot of things right on the Internet. Many people have their own blogs. They also create some videos on YouTube or short funny videos on some type of social media, or they make funny pictures for social media. So, a lot of people also give something to the Internet, not only take it from it.”*

#### 3.2.1. I know

The first theme, “I know,” focused on the role of technology as a *source of information* and on *formal and non-formal education*. This was regarded as the most frequently mentioned benefit by adolescents and was associated with the knowledge they could gain *via* digital technologies or the Internet. Adolescents described the Internet as a *huge source of information* that offered them the opportunity to find everything they need, which they considered very helpful, including in their preparation for school. Moreover, knowing that they had access to such a source of information gave them a sense of freedom.

Furthermore, adolescents described digital technology as a tool that can be used to provide *online formal and non-formal education*, especially during the pandemic. They reported many previous experiences with different kinds of applications, online quizzes, and tests available on the Internet that helped them improve their knowledge in the subjects they studied at school, for example, language or chemistry. The internet can also be used as an educational aid by teachers in the classroom, so as to make learning more fun and interesting.

#### 3.2.2. I can

Regarding the theme “I can,” adolescents expressed some benefits linked to the features of digital technology. They described digital technology as a *smart device*, characterized by compactness or multi-functionality, that gave them the feeling of having everything they needed at their fingertips.

Furthermore, they found digital technology to be a very *helpful tool*, thanks, especially to all the applications that it provides. On the one hand, it helped them get oriented in time or space, thanks to calendars, watches, and maps. On the other hand, they reported that it can serve as a repository for their own thoughts, ideas, or important materials, which they can access at any time. Moreover, the Internet is not only considered useful in crises, as it offers quick access to the contact information of different helplines, but also in the process of job-seeking.

#### 3.2.3. I am connected/I am included

Most adolescents believe that thanks to digital technology they can “be connected or included,” providing the title for this theme, which includes *social interactions* as another suggested benefit. The most frequently mentioned advantages by adolescents in this context included the ability to connect with others and the easy way to communicate or build/maintain their relationships. Digital technology can serve as a helpful tool in the development of their social interactions, as it facilitates communication or experience-sharing with friends or family members, especially those living abroad. It made those people more available to them. Moreover, adolescents admitted that the Internet helped them feed their need to be updated. Not only can technology play a crucial role by increasing the frequency of contacts, but it can also enhance the quality of existing relationships. Adolescents found technology extremely beneficial for making some relationships deeper, for example, with their teachers or people who do not prefer face-to-face communication. Another benefit suggested by adolescents was that technology provided a free and fast way to organize different kinds of formal and non-formal meetings.

#### 3.2.4. I have my comfortable place

Adolescents described digital technology as being a place where “they have their comfortable world.” They mentioned two benefits associated with this theme: *creating their alternative world* and engaging in *leisure activities*. Some adolescents perceived digital technology as a tool that helps them *create their own alternative world*, which they can tailor to their own needs or interests. It is a place where they can feel good or happy, as they have the opportunity to choose the things that will surround them in comparison to the offline world, by following the people who match their preferences and who can motivate them to engage in some activity or achieve their goals.

In addition, digital technology provides adolescents with an easy and fast way to find *leisure activities*. More specifically, they perceive the Internet as a place where they can unwind or just switch off for a bit. A dominant proportion of adolescents admitted to connecting to the Internet during school preparation breaks or just to fill idle time. They mentioned several activities that served them for this purpose, such as browsing social media, reading online books, watching movies, listening to music, or playing games, which supported their social lives.

#### 3.2.5. I work on my future

Lastly, adolescents perceived *self-development* as an important benefit related to digital technology; this relates to our last theme, “I work on my future.” The most frequently mentioned benefits by adolescents in this context were gaining motivation, self-improvement in existing hobbies, and sharing their skills. Adolescents believe that digital technology and the Internet offer them ideas or inspiration that can motivate them to start doing some kind of activity. They admitted that sometimes only watching people perform some activity could already work as stimulus. Furthermore, adolescents can find many tutorials or instructions shared by other users on the Internet that can help them develop or improve their hobbies. They also perceive the Internet as a place that gives them the opportunity to show off their talent or skills, for example, by writing blogs where they can share their thoughts or opinions or by publishing different kinds of videos on their YouTube channel. Moreover, they reported that the Internet can play a crucial role in promoting important topics or the activities of organizations for which it is very difficult to get help, such as “Homeless is more.”

## 4. Discussion

Our study aimed to explore the benefits of digital technology based on adolescents’ subjective perceptions and experiences. We identified five main themes in their statements: the adolescents perceived digital technology as a smart, helpful device *(I can)*; a source of information supporting their formal or non-formal education *(I know)*; a means of developing their social interactions *(I am connected/I am included)*; a means of participating in leisure time activities, creating their alternative world *(I have my comfortable world)*; and a means of working on their self-development *(I work on my future)*.

### 4.1. I know

We found that adolescents viewed digital technology as a very useful educational tool, not only in school settings but also because it provides a vast amount of information. This is in line with the findings of previous studies on this topic (Kibirige and DePalo, 2000; Dogruer et al., 2011; Baheiraei et al., 2014; Shonola et al., 2016). For example, Dogruer et al. (2011) showed that adolescents feel particularly at ease using search tools or social media, which can serve as a place for discussions about school subjects and help them clarify their uncertainties about topics. This finding can be explained as follows: today’s adolescents prefer to use online resources for information, mostly because of their easy and fast accessibility (Lupton, 2021). More specifically, thanks to an Internet connection, they can obtain copious amounts of data or different perspectives on a topic in a relatively short time and without going anywhere. Moreover, thanks to this technology, they have easy access to online school materials shared by teachers, and this allows them to engage in learning activities even if they do not attend their classes. This can also be very useful in improving self-study flexibility (Shonola et al., 2016).

Additionally, research has shown that the context of video games often motivates adolescents to learn about different topics that they encounter at school; e.g., adolescents used information from a game – Minecraft (a survival-based game focused on discovering and gathering different resources, such as raw materials for craft tools to build the world and stay alive) – in physics or chemistry class. Many adolescents confirmed that playing video games helped them improve their language skills more than the English lessons at school (Pereira et al., 2019). Thanks to video games, they could meet players from different countries of the world, forcing them to use English if they wanted to talk, for instance, about game strategy. This can then highly supplement what they learn at school; language teachers’ pay more attention to teaching their students about grammar than to developing their conversation skills. In addition, adolescents’ responses confirmed the positive impact of computer games on their cognitive skills, too, such as reasoning or visual processing (Subrahmanyam et al., 2000; Pereira et al., 2019). Thus, we can conclude that digital technology and its applications give adolescents the possibility to gain knowledge or skills outside of formal education, allowing them to become independent learners who can choose what they want to study and how they want to study it (Sivalingam and Subbaiyan, 2018).

### 4.2. I can

From the perspective of adolescents, the instrumental features of digital technology can contribute to what they can do. Adolescents reported that digital technology is a smart and helpful device thanks to which they can get orientated in time or to a place. This finding is in line with the study conducted by Ling and Yttri (2002), which suggests that one of the most important functions of digital technology is that it supports micro-coordination, i.e., organizing the everyday lives of users, thanks to which they feel more flexible in terms of time and location.

Additionally, adolescents who participated in the study reported that digital technology can serve as a quick way to access different types of help. This finding is consistent with previous research showing that digital technology provides adolescents with a higher sense of security and plays a role in empowering them not only in emergencies but also in situations that some people may perceive as stressful, such as visiting public spaces (Pain et al., 2005; Tennakoon and Taras, 2012). It also aligns with the finding that many adolescents tend to turn to online counseling platforms or applications in crises, as they provide them with a space where they can feel emotionally safe and talk easier about their problems due to the distance between them and their counselors. This kind of service allows them to remain anonymous, maintain their privacy, avoid embarrassment when discussing their problems in person, and reduce the potential feeling of stigma (Pretorius et al., 2019; Wong et al., 2021). Another benefit of such help is that it is also accessible at times when formal services are not available, e.g., at night (Lásková, 2016; Pretorius et al., 2019). Moreover, the use of online help resources also helps meet users’ desires to retain self-reliance. In the online setting, they still have a chance to decide if they will accept the proposed advice or deal with their problems by themselves. Finally, a study by O’Dea et al. (2020) showed that help apps can be very useful in supporting adolescents’ intentions to seek help by showing them that their situation requires professional help, presenting them with coping strategies that could be effective for their problems, or reassuring them that it is normal to ask for help. Thus, giving a mobile phone to adolescents can also be perceived by parents as a way to keep their children safer and healthier (Williams and Williams, 2005). In such a situation, digital technology can be beneficial not only for adolescents but also for their parents.

### 4.3. I am included/connected

Next, the interviewed adolescents reported that digital technology can play a key role in the development of their social lives. Our findings are in line with research showing that although online communication led to a decrease in adolescents’ face-to-face interaction with parents or friends, this did not affect the quality of those relationships (Lee, 2009). In addition, it strengthened their social ties with friends, which positively affected their well-being (Valkenburg and Peter, 2007; Lee, 2009). This can be explained by the higher level of controllability linked to online communication, which can encourage adolescents to open up to others, thus increasing the quality of their relationships (Joinson, 2001; Valkenburg and Peter, 2007). Moreover, digital technology can be very useful even for those adolescents with fewer social skills. Even introverted adolescents find it easier to self-disclose in the digital world, where they can choose what personal information to share (Peter et al., 2005). Digital technology also has a huge potential to include people who are disadvantaged due to reasons such as poverty, crisis, or disease. On the one hand, it allows them to get support from or connect with other people who are facing similar life conditions. On the other hand, it can link them to opportunities, such as online education and job offers, that are available to all people without discrimination (UNICEF for Every Child, 2017; Rideout and Fox, 2018). Thus, digital technology gives adolescents the possibility to feel more equal thanks to its capacity to connect people from different socioeconomic backgrounds, countries, or personalities.

### 4.4. I have my comfortable world

We found that, from the perspective of adolescents, digital technology gives them the option to create their own comfortable world, tailored to their needs and interests. They can choose the kind of content that will be presented to them when they go online in the digital world, for example, by following people who motivated them in doing some activity or achieve their goals. The theme of creating an alternative world seems to be understudied, but some investigations have explored related topics (Djafarova and Rushworth, 2017; Croes and Bartels, 2021). They have shown that adolescents tend to follow social influencers to relax and have fun or to get information about products that are interesting to them (Djafarova and Rushworth, 2017; Croes and Bartels, 2021). Social influencers try to get closer to their fans by communicating with them. Therefore, adolescents can have the feeling that they are more accessible to them, just like their activities or achievements, which can ultimately motivate them in their own lives. This may be one reason why they chose them to be a part of their comfortable world.

Additionally, we found that many adolescents perceive online leisure activities as an important benefit of digital technology, as these allow them to switch off a bit. This is consistent with Khan’s (2017) findings that adolescents often browse social media or watch videos passively because of a need to relax or be entertained. Furthermore, Cheah et al. (2021) suggested other motives that may lead to playing digital games, such as immersion/flow, gratification/affect, escapism, social interaction, identification, and goal orientation. The research of Helstrom et al. (2015) revealed that escape motives or playing to gain status in combination with excessive gaming time can lead to health problems such as depressive or musculoskeletal symptoms. Therefore, we can conclude that spending time in online activities can be beneficial for adolescents by providing them with a quick access to fun, relaxation, or friends, but it is very important to always consider the motives for this behavior, as these play a key role in determining its ultimate impact on adolescents’ lives (Subrahmanyam et al., 2000).

### 4.5. I work on my future

We found that adolescents view digital technology as the place where they can work on their future. They suggested that it serves as a platform where not only they can learn something, but also display a part of themselves or their talent. This finding is in line with studies showing that public websites such as YouTube and MySpace, and even interests-driven online communities (such as writing groups) based on peer reciprocity can be very useful tools for the development of adolescents in the future (Iftikhar et al., 2019). On the one hand, this type of virtual place gives them the possibility to share their own experience or work/talent with a broader audience or with people who are important in their relevant field; thus, they can get constructive feedback that can help them improve (Ito et al., 2010; Iftikhar et al., 2019). On the other hand, they have the opportunity to view the experiences of others, which can provide them with inspiration or social support when engaging in a particular activity (Radovic et al., 2018). Specifically, if they are struggling in the beginning, seeing the path of others to success and everything that comes with it can make them feel they are not alone, which can encourage them to continue working on themselves. We can thus conclude that gaining a reputation, and fans, receiving constructive feedback on creative work, and seeing the experiences of others can also play a significant role in increasing adolescents’ self-confidence, in addition to providing more motivation.

## Strengths and limitations

A major strength of this study is its qualitative design, which allowed us to provide detailed insight into adolescents’ subjective perceptions and experiences on the benefits of digital technology based on their own statements, which have been video-recorded, transcribed verbatim, and reviewed by all team members before we proceeded to the analysis. In addition, thanks to the consensual qualitative research methodology, we surpassed the subjective perspectives of the researchers, as all the coders had to reach a consensus regarding the codes used for the analyzed data. Lastly, our study presents adolescents’ perceptions of the benefits of digital technology in all areas of life, whereas previous studies on this topic focused mainly on its risks or advantages in social life.

Our study is limited by the relatively small and not fully heterogeneous sample with respect to gender and secondary school type. This limitation may be attributable to the fact that our interviews were conducted during the second wave of the COVID-19 pandemic in Slovakia, and due to government measures, we did not have the chance to meet with participants in schools, which may have resulted in reduced participation in our study. However, we reached saturation during data collection based on this sample, i.e., no new themes emerged during the last interviews. A second limitation may be that, because of COVID-19, we conducted our study dedicated to the topic of digital technology *via* an online platform, which may have led to an information bias. We tried to reduce the likelihood of this bias by using open-ended questions that asked about adolescents´ personal experiences or subjective perspectives regarding digital technology.

## Implications

Digital technology has been shown to have positive meaning for adolescents, as it facilitates their lives in many ways, for example, regarding their school environment (applications for the educational process, platforms useful for self-development). This implies a greater focus on the appropriate use of digital technology in school settings, which is also contingent upon the media literacy level of teachers. Therefore, educational policies should prioritize integrating continuous training plans for teachers in this area. In today’s society, a negative perception of digital technology or the Internet is prevalent; however, these technologies are inevitable and therefore need to be used in an adequate manner in terms of time spent and user safety. A more positive perception of digital technology and its opportunities may lead to greater adoption in a variety of societal domains.

Our study is one of the first to confirm that digital technology can play a crucial supportive role in different life domains if it is used in an appropriate way. These findings should be confirmed by more studies on different age groups, and the benefits regarding areas other than social life. The exploration of needs met by adolescents through digital technology enables us to investigate aspects of the offline world that need to be strengthened. In addition, further research should identify the groups of adolescents who are less likely to benefit from digital technology and how technology can be made more useful for them. This may provide important information not only for its future development but also for enhancing adolescents’ adaptation to its demands. Moreover, future research can focus on the exploration of the effect of digital technology on the transition to adulthood and the adaptation to adult roles as part of mental and social health.

## Conclusion

Our findings show that digital technology can support adolescents in various domains of their lives and that they are aware of the opportunities regarding its use. This demonstrates that putting adolescents and their opinions at the center of national and even global digital policies can be very helpful, not only in reducing their exposure to the risks associated with the technology but also in exploring how it can be useful in realizing their potential (UNICEF for Every Child, 2017). Moreover, our research suggests that digital technology can enable positive personal growth, self-perception, and mental well-being in adolescents. Although more research is focused on the risks of digital technology, adolescents’ presence in the online world can also support positive adaptation to this developmental period, as well as their socialization as opposed to isolation.

## Data availability statement

The raw data supporting the conclusions of this article will be made available by the authors, without undue reservation.

## Ethics statement

The studies involving human participants were reviewed and approved by Ethics Committee of the Medical Faculty at the Pavol Jozef Safarik University in Kosice (19 N/2020). Written informed consent to participate in this study was provided by the participants’ legal guardian/next of kin.

## Author contributions

LBU, AMG, ZDV, and SC participated in the design and coordination of the study and data collection. LBU conducted literature searches and provided summaries of previous research. LBU, AMG, ZDV, and SC worked on the analyses and interpretation of the data. LBU drafted the initial manuscript, and AMG, ZDV, SC, JH, JPD, and SAR provided supervision, contributed their comments to the manuscript, and approved its final version, as submitted. All authors contributed to the article and approved the submitted version.

## Funding

This work was supported by the Slovak Research and Development Agency under Contract No. APVV-18-0070.

## Conflict of interest

The authors declare that the research was conducted in the absence of any commercial or financial relationships that could be construed as a potential conflict of interest.

## Publisher’s note

All claims expressed in this article are solely those of the authors and do not necessarily represent those of their affiliated organizations, or those of the publisher, the editors and the reviewers. Any product that may be evaluated in this article, or claim that may be made by its manufacturer, is not guaranteed or endorsed by the publisher.
